# Robust selenium-doped carbon nitride nanotubes for selective electrocatalytic oxidation of furan compounds to maleic acid†

**DOI:** 10.1039/d1sc01231b

**Published:** 2021-04-01

**Authors:** Xin Huang, Jinliang Song, Manli Hua, Bingfeng Chen, Zhenbing Xie, Huizhen Liu, Zhanrong Zhang, Qinglei Meng, Buxing Han

**Affiliations:** Beijing National Laboratory for Molecular Science, CAS Key Laboratory of Colloid and Interface and Thermodynamics, CAS Research/Education Center for Excellence in Molecular Sciences, Institute of Chemistry, Chinese Academy of Sciences Beijing 100190 China songjl@iccas.ac.cn hanbx@icas.ac.cn; School of Chemistry and Chemical Engineering, University of Chinese Academy of Sciences Beijing 100049 China; Physical Science Laboratory, Huairou National Comprehensive Science Center Beijing 101400 China

## Abstract

Selective oxidation of biomass-derived furan compounds to maleic acid (MA), an important bulk chemical, is a very attractive strategy for biomass transformation. However, achieving a high MA selectivity remains a great challenge. Herein, we for the first time successfully designed and fabricated Se-doped graphitic carbon nitride nanotubes with a chemical formula of C_3.0_N-Se_0.03_. The prepared C_3.0_N-Se_0.03_ was highly efficient for electrocatalytic oxidation of various biomass-derived furan compounds to generate MA. At ambient conditions, the MA yield could reach 84.2% from the electro-oxidation of furfural. Notably, the substituents on the furan ring significantly affected the selectivity to MA, following the order: carboxyl group > aldehyde group > hydroxyl group. Detailed investigation revealed that Se doping could tune the chemical structure of the materials (*e.g.*, C_3.0_N-Se_0.03_ and g-C_3_N_4_), thus resulting in the change in catalytic mechanism. The excellent performance of C_3.0_N-Se_0.03_ originated from the suitable amount of graphitic N and its better electrochemical properties, which significantly boosted the oxidation pathway to MA. This work provides a robust and selective metal-free electrocatalyst for the sustainable synthesis of MA from oxidation of biomass-derived furan compounds.

## Abstract 2

Biomass-derived furan compounds could be selectively electro-oxidized into maleic acid over a robust selenium-doped carbon nitride nanotube catalyst.

## Graphical abstract

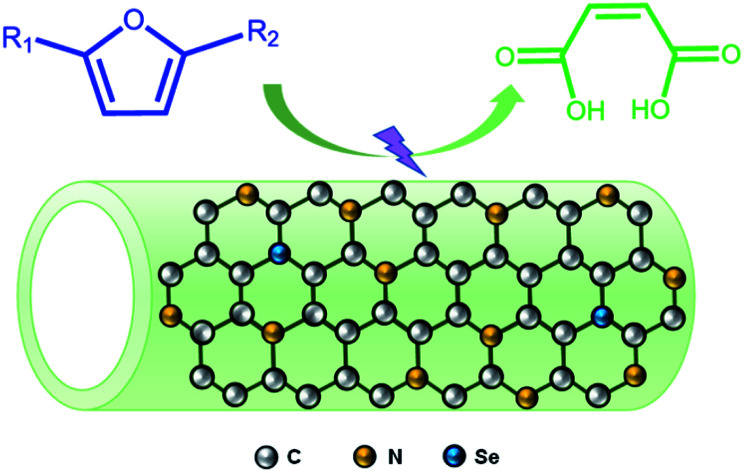

## Introduction

Maleic acid (MA) is a versatile bulk chemical, and can be used as the critical raw material or intermediate to manufacture unsaturated polyester resins, plasticizers, surface coatings, and pharmaceuticals.^1^ Currently, the majority of MA is produced by the catalytic oxidation of petroleum-based chemicals (*i.e.*, *n*-butane,^2^ butenes,^3^ benzene and its derivatives^4^) with the annual output of millions of tons. To alleviate excessive reliance on the diminishing fossil resources, the production of useful commercial chemicals from renewable and abundantly available lignocellulose has triggered significant interest in the past decade.^5^ In this regard, partial oxidation of lignocellulose-derived furan compounds (*e.g.*, furfural and 5-hydroxymethylfurfural) has been considered as a promising and sustainable strategy for the production of MA.^6^

At the early stage, MA could be produced with the yield of about 70% *via* the vapor-phase oxidation of furfural.^7^ However, the elevated temperature (above 200 °C) was the major limitation of this process. Lately, researchers developed the liquid-phase oxidation of furfural and 5-hydroxymethylfurfural for the synthesis of MA in various catalytic systems.^8^ In spite of the achieved progress, the MA yield in most of the developed liquid-phase systems could hardly exceed 60% because of the unstable nature of furfural and 5-hydroxymethylfurfural, and complicated and harsh conditions were generally needed, including high temperature and O_2_ pressure, the employment of chemical oxidants, or the use of organic solvents (especially organic carboxylic acid).

It is known that electrocatalysis is a promising route to reach mild and selective organic reactions.^9^ Occurring at ambient conditions and excluding the use of chemical oxidants, electrocatalytic synthesis of MA from lignocellulose-derived furan compounds is expected to become a competitive strategy for future MA production. It has been reported that MA could be produced from electrocatalytic oxidation, but toxic PbO_2_ was employed as the electrode and the MA yield was only 65.1%.^10^ Thus, robust and low-cost electrode materials are still highly desirable for the synthesis of MA *via* electrocatalytic oxidation of lignocellulose-derived furan compounds into MA.

With the advantages of low cost, high electrical conductivity, and easy turnability in catalytic activity, metal-free carbon-based materials have been employed as advanced electrode materials in different electrochemical processes.^11^ However, the utilization of metal-free carbon-based electrocatalysts for biomass conversion is still largely limited owing to the high overpotential, low catalytic activity and product selectivity. Generally, the electrocatalytic performance of carbon-based materials can be finely boosted through the construction of advanced nanostructures or the doping of heteroatoms. It is of significant attraction to develop advanced metal-free carbon-based materials for electrocatalytic conversion of biomass.

To realize efficient synthesis of MA from furan compounds *via* electrocatalytic oxidation, herein, we for the first time fabricated the selenium (Se)-doped graphitic carbon nitride nanotubes with the C/N and Se/N atomic ratios of 3.0 and 0.03, respectively, named as C_3.0_N-Se_0.03_. The synthesized C_3.0_N-Se_0.03_ as a metal-free catalyst showed superior electrocatalytic activity on the oxidation of renewable furan compounds into MA. At ambient conditions, furfural could be selectively converted into MA with a yield of 84.2% in 0.5 M KHCO_3_ aqueous solution, which was much higher than those in the reported typical heterogeneous systems (Table S1†). Notably, when the furan compounds became furoic acid and 2,5-furandicarboxylic acid, which could be easily achieved from electrocatalytic oxidation of renewable furfural and 5-hydroxymethylfurfural,^12^ very high yields (>90%) of MA could be obtained, which has never been realized previously. The high selectivity at ambient conditions and the wide applicability confirmed the great potential of the C_3.0_N-Se_0.03_ on the electrocatalytic oxidation of furan compounds to synthesize MA.

## Results and discussion

### Preparation and characterization of the C_3.0_N-Se_0.03_ nanotubes

**Fig. 1:**
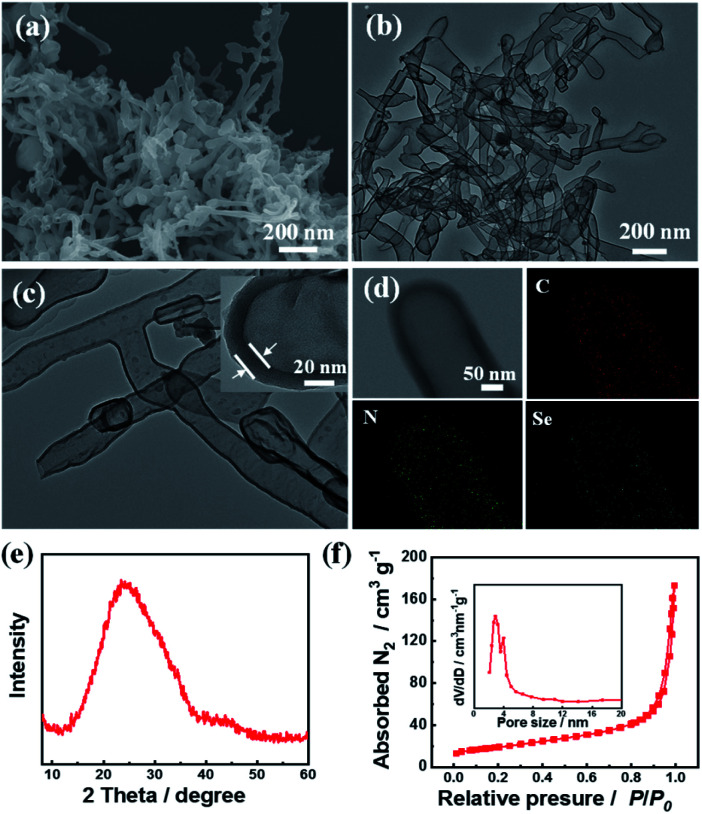
Characterizations of the prepared C_3.0_N-Se_0.03_. (a) SEM image, (b) TEM image, (c) HR-TEM image, (d) elemental mappings, (e) XRD pattern, and (f) N_2_ adsorption–desorption isotherm.

The target material (C_3.0_N-Se_0.03_ nanotubes) was prepared by pyrolyzing the precursors of urea, NH_4_Cl, and SeO_2_, and the detail process was described in ESI.† Characterized by SEM (Fig. 1a), the C_3.0_N-Se_0.03_ had a rod-like nanostructure with the length of 200 to 1000 nm. The nanotube structure could be further verified by TEM (Fig. 1b), and the wall thickness of the C_3.0_N-Se_0.03_ nanotubes was about 20 nm with the interior diameter of about 150 nm (Fig. 1c). EDX mapping of C_3.0_N-Se_0.03_ showed that the elements of C, N and Se were distributed uniformly on the wall of the nanotube (Fig. 1d). In the XRD pattern of C_3.0_N-Se_0.03_ (Fig. 1e), a single broad diffraction peak at 24.8° was found, implying that the C and N in C_3.0_N-Se_0.03_ were arranged in an ordering turbostratic form,^13^ which was essential for redox reactions.^14^ Meanwhile, no peaks for SeO_2_ or NH_4_Cl were found in the pattern, verifying the complete volatilization of these species during the preparation process of C_3.0_N-Se_0.03_. As determined by N_2_ adsorption-desorption method, the C_3.0_N-Se_0.03_ possessed a well-defined mesoporous structure (Fig. 1f), and the BET surface area of C_3.0_N-Se_0.03_ was 67.1 m^2^ g^−1^. Additionally, the Se content determined by ICP-MS was 2.39 wt% in the C_3.0_N-Se_0.03_, while the C/N atomic ratio was 3.0 based on the element analysis.

**Fig. 2:**
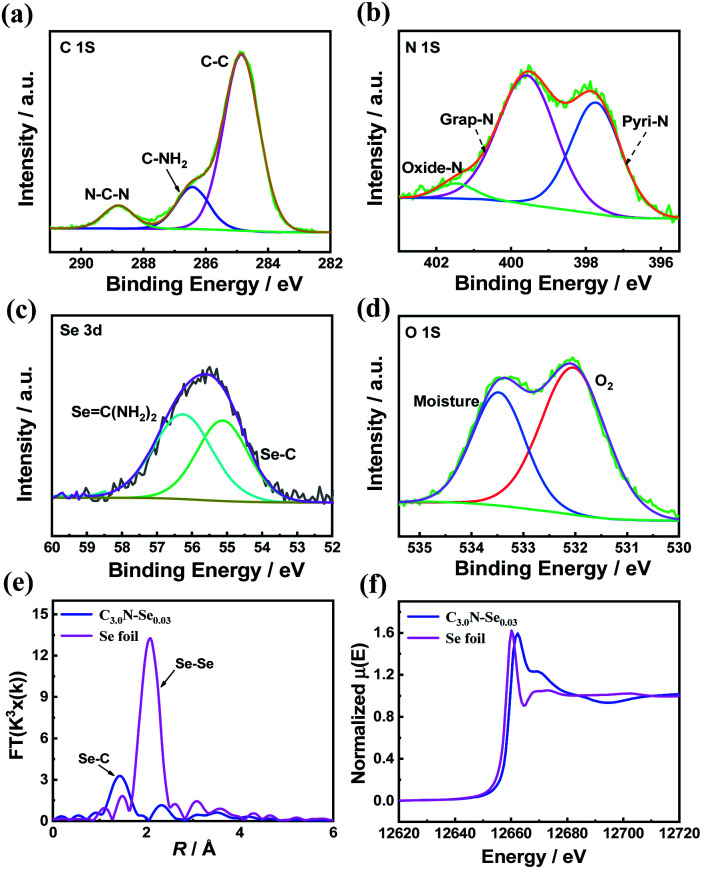
Chemical structure characterization of the C_3.0_N-Se_0.03_. (a) High-resolution XPS spectra of C 1s, (b) high-resolution XPS spectra of N 1s, (c) XPS spectra of Se 3d, (d) XPS spectra of O 1s, (e) Se K-edge Fourier transformed EXAFS spectra in the R space, and (f) Se K-edge XANES.

The fine structure of C_3.0_N-Se_0.03_ was further characterized by Raman spectrum and X-ray photoelectron spectroscopy (XPS). Raman spectrum of C_3.0_N-Se_0.03_ showed intense D band at 1340 cm^−1^ and the G band at 1590 cm^−1^ (Fig. S1†), indicating the well-defined graphene-like structure of C_3.0_N-Se_0.03_. Furthermore, the surface chemical composition of the C_3.0_N-Se_0.03_ was determined by XPS technique. XPS survey spectrum of the C_3.0_N-Se_0.03_ (Fig. S2†) could further confirm the presence of C, N, O, and Se, and the absence of Cl, proving the complete volatilization of NH_4_Cl. High-resolution XPS spectra of C 1s indicated that there were three carbon species with the corresponding binding energies at 284.7, 286.4 and 288.7 eV (Fig. 2a), which attributed to sp^2^ C–C in the framework, C–NH_2_ at the edges and sp^2^ C–N–C groups, respectively.^15^ The signal of N 1s could be divided into three peaks at 397.7, 399.8, and 401.5 eV (Fig. 2b), which corresponded to the pyridinic N, graphitic N, and oxidized N, respectively.^16^ Furthermore, for Se 3d, two peaks at 55.4 and 56.3 eV, which were assigned to Se=C–N and Se–C bonds, could be observed (Fig. 2c), implying that Se atoms were incorporated into the framework of the C_3.0_N-Se_0.03_.^17^ Additionally, weak O 1s signal was assigned to surface adsorbed adventitious oxygen-containing species (Fig. 2d). The Fourier-transformed (FT) *k*_3_-weighted extended X-ray absorption fine structure (EXAFS) showed that there was only Se–C coordination peak (at 1.44 Å), and no Se–Se coordination peak (at about 2.08 Å) was found (Fig. 2e),^18^ further verifying that Se element was bonded by C and atomically dispersed throughout the C_3.0_N-Se_0.03_ framework. Se K-edge X-ray absorption near-edge structure (XANES) showed that the absorption edge of C_3.0_N-Se_0.03_ located slightly higher energy position than that of Se foil (Fig. 2f), indicating the valence state of Se atom in framework is higher than 0.^19^

In comparison, several other C_*x*_N-Se_*y*_ materials were synthesized at mass ratios of SeO_2_ to urea of 0.1, 0.25, 0.5, and 1.5, respectively. Based on the C/N and Se/N ratios, these materials were named as C_0.7_N-Se_0.002_, C_0.9_N-Se_0.003_, C_2.1_N-Se_0.01_, and C_4.2_N-Se_0.05_. Meanwhile, g-C_3_N_4_ (synthesized from urea) and g-C_3_N_4_-AC (synthesized from urea and NH_4_Cl) were also prepared. The characterizations (TEM, SEM, XRD, Raman spectra, and XPS) of these materials were described in the ESI (Fig. S3–S11†). From the characterizations, the SeO_2_ amount could significantly affect the morphology and chemical structure of the obtained materials. With the increase of SeO_2_ amount, the morphology changed from nanosheets (g-C_3_N_4_, g-C_3_N_4_-AC, C_0.7_N-Se_0.002_, and C_0.9_N-Se_0.003_) to nanotubes (C_3.0_N-Se_0.03_ and C_4.2_N-Se_0.05_), while the chemical structure changed from the tri-*s*-triazine structure into graphitic carbon nitride. These results implied that SeO_2_ played the template role for the formation of graphitic carbon nitride nanotubes.

### Electrocatalytic performances for furfural oxidation

**Fig. 3:**
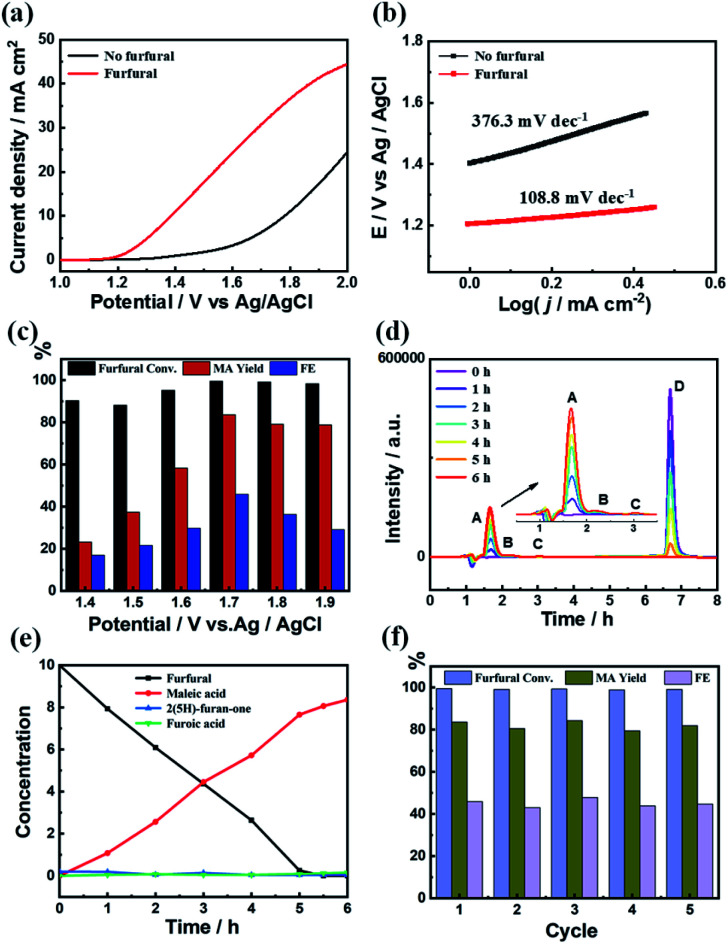
(a) LSV curves of C_3.0_N-Se_0.03_ at a scan rate of 50 mV s^−1^ in 0.5 M aqueous KHCO_3_ solution, (b) Tafel plots of C_3.0_N-Se_0.03_ with and without furfural, (c) the furfural conversion, MA yield and FE of MA at different applied potentials over C_3.0_N-Se_0.03_ electrode, (d) HPLC chromatogram at different electrolysis times (A, B, C and D represent for MA, 2(5*H*)-furanone, FA and furfural, respectively), (e) concentrations of furfural and the oxidation products at various electrolysis times, and (f) reusability of C_3.0_N-Se_0.03_ electrode. Reaction conditions: applied potential, 1.7 V *vs.* Ag/AgCl; furfural concentration, 10 mM; 0.5 M aqueous KHCO_3_ electrolyte, 15 mL; reaction time, 6 h.

The electrocatalytic performance of the C_3.0_N-Se_0.03_ material on furfural oxidation was conducted in a three-electrode electrolysis cell employing aqueous KHCO_3_ solution (0.5 M) as the electrolyte (both anolyte and catholyte). As shown in Fig. 3a, the water oxidation occurred at an onset potential of about 1.3 V *vs.* Ag/AgCl without furfural. After adding furfural (10 mM), the onset potential shifted to 1.2 V *vs.* Ag/AgCl accompanied by a rapid increase of current density, implying the occurrence of furfural oxidation on the C_3.0_N-Se_0.03_ electrode. Furthermore, the Tafel slope significantly decreased from 376.3 mV dec^−1^ in the absence of furfural to 108.8 mV dec^−1^ in the presence of furfural (Fig. 3b), indicating a much faster catalytic kinetics after adding furfural.

**Fig. 4:**
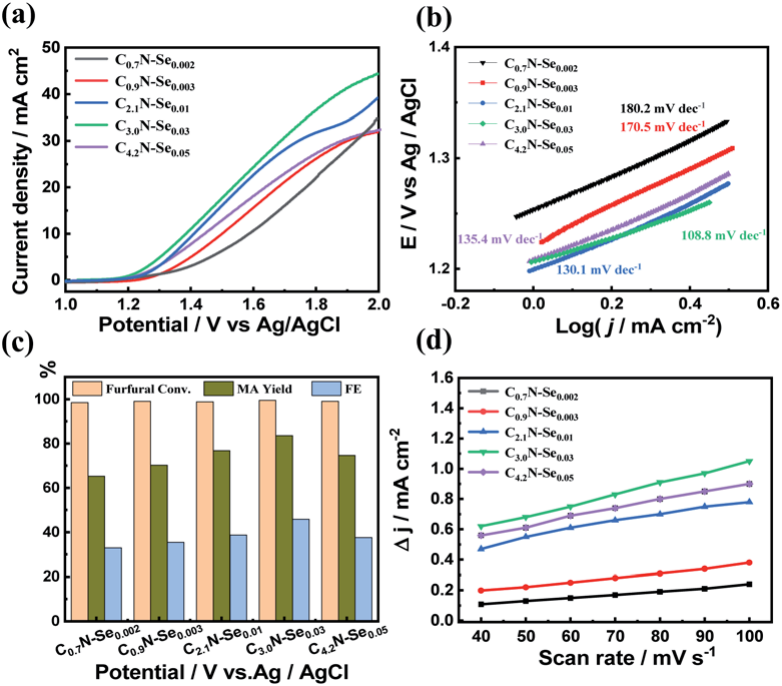
(a) LSV curves of a serious of C_*x*_N-Se_*y*_ electrodes at a scan rate of 50 mV s^−1^ with 10 mM furfural in 0.5 M KHCO_3_ solution, (b) Tafel plots of C_*x*_N-Se_*y*_ electrodes with furfural, (c) the furfural conversion, MA yield and FE of MA over C_*x*_N-Se_*y*_ electrodes at 1.7 V *vs.* Ag/AgCl, and (d) charging current density differences plotted against scan rates for C_*x*_N-Se_*y*_ electrodes.

As is well-known, the applied potentials generally have significant impact on the results. As shown in Fig. 3c, the yield and faradaic efficiency of MA increased with the increase of the applied potentials at first (1.4–1.7 V *vs.* Ag/AgCl) and then decreased at the applied potentials higher than 1.7 V *vs.* Ag/AgCl. Meanwhile, although the conversions of furfural were high at the applied potentials below 1.7 V *vs.* Ag/AgCl, the yields of MA were low with low yields of 2(5*H*)-furanone and furoic acid (Fig. S12a†), which was caused by the undesired side-reactions of furfural. These results indicated that the optimal potential was 1.7 V *vs.* Ag/AgCl over C_3.0_N-Se_0.03_ in 0.5 M KHCO_3_ electrolyte. Moreover, as monitored by high-performance liquid chromatography (HPLC), the added furfural could be completely consumed in 6 h at the applied potential of 1.7 V *vs.* Ag/AgCl (Fig. 3d and e), while the yield of MA continually increased and reached the maximum value of 84.2% in 6 h with a faradaic efficiency of 45.9%. Besides, the current density slightly decreased during the reaction process because the added furfural was consumed (Fig. S12b†). More importantly, the C_3.0_N-Se_0.03_ electrode could be reused for five cycles with no obvious decrease in the MA yield and the faradaic efficiency (Fig. 4f). No notable difference for XPS spectra was observed for the fresh and recovered C_3.0_N-Se_0.03_ (Fig. S13†), suggesting the high stability of C_3.0_N-Se_0.03_ electrode under the reaction conditions.

In another aspect, the property of the used electrolytes could affect the electrocatalytic performance of the C_3.0_N-Se_0.03_. As showed in the LSV curves (Fig. S14†), higher current density and lower onset potential could be achieved in the experiment with furfural (10 mM) than those without furfural in all examined electrolytes (*i.e.*, 0.5 M (NH_4_)_2_SO_4_, 1 M KOH, 0.5 M H_2_SO_4_, and phosphate buffered solution), suggesting that furfural could be oxidized in all these electrolytes. From Table S2,† we could find that the conversions of furfural were high in all the examined electrolytes. However, the yield of the desired MA varied with the electrolytes. It was obvious that weak basic electrolytes (0.5 M KHCO_3_) were beneficial for the generation of MA, while the acidic (0.5 M H_2_SO_4_ and 0.5 M (NH_4_)_2_SO_4_), neutral (phosphate buffered solution) and strong basic (1 M KOH) electrolytes provided very low yields of MA owing to undesired side-reactions of furfural in these electrolytes. Based on the results in Table S2,† 0.5 M KHCO_3_ was employed as the optimal electrolyte over C_3.0_N-Se_0.03_.

### Activity of g-C_3_N_4_ and various C_*x*_N-Se_*y*_ materials

The activity of C_3.0_N-Se_0.03_ and g-C_3_N_4_ was initially compared. Although the current density also increased after the furfural was added over g-C_3_N_4_ (Fig. S15a†), the increased degree was lower than that over C_3.0_N-Se_0.03_, implying that C_3.0_N-Se_0.03_ was more active for the electrocatalytic oxidation of furfural. Indeed, the reaction time was 12 h over g-C_3_N_4_ when the furfural was completely converted at an optimized applied potential of 1.8 V *vs.* Ag/AgCl, and the yield of MA was only 55.2% (Fig. S16†).

Subsequently, the electrocatalytic performance of various C_*x*_N-Se_*y*_ materials was studied in 0.5 M KHCO_3_ electrolyte. Similarly, furfural oxidation could occur over these materials because the current density was increased and the onset potential was lowered when furfural was added (Fig. 4a and S17†). Among these C_*x*_N-Se_*y*_ materials, C_3.0_N-Se_0.03_ yielded the highest current density and the lowest onset potential with furfural (Fig. 4a), indicating that it was more active for the furfural oxidation. When the reaction was conducted at 1.7 V *vs.* Ag/AgCl (Fig. 4c), the MA yield increased with the increase of the C/N atomic ratio and the Se amount, and C_3.0_N-Se_0.03_ provided the highest MA yield (84.2%). However, the MA yield decreased when the C/N atomic ratio was increased to 4.2. These results indicated that the electrocatalytic performance of the C_*x*_N-Se_*y*_ materials for furfural oxidation was significantly affected by the amount of Se and the C/N atomic ratio.

In order to compare the kinetics of furfural oxidation among various C_*x*_N-Se_*y*_ materials, Tafel and the electrochemical active surface area (ECSA) analysis was performed. First, from the LSV curves, the Tafel slope of C_3.0_N-Se_0.03_ (108.8 mV dec^−1^) was much lower than that of g-C_3_N_4_ (263.8 mV dec^−1^, Fig. S15b†), and was the lowest among various C_*x*_N-Se_*y*_ materials (Fig. 4b), suggesting its high intrinsic electrocatalytic activity for furfural oxidation into MA. Second, C_3.0_N-Se_0.03_ showed much higher electrochemical surface area (ECSA) than g-C_3_N_4_ (Fig. S18a†), and had the highest ECSA among the C_*x*_N-Se_*y*_ materials (Fig. 4d), indicating C_3.0_N-Se_0.03_ had more catalytically active sites for furfural oxidation, which favored the reaction. Third, the charge transfer resistance (*R*_ct_) of C_3.0_N-Se_0.03_ was lower than that of g-C_3_N_4_ (Fig. S18b†) and other C_*x*_N-Se_*y*_ materials (Fig. S19†), suggesting a more facile electron transfer process over C_3.0_N-Se_0.03_ to promote the reaction. On the basis of the above discussions, C_3.0_N-Se_0.03_ had the highest electrochemical surface area and lowest charge transfer resistance among the examined metal-free carbon-based materials (*i.e.*, g-C_3_N_4_ and C_*x*_N-Se_*y*_). These advantages could significantly improve the oxidation efficiency of furfural to generate MA, and thus decrease the occurrence of the side-reactions. Thereby, a much higher selectivity of MA could be achieved over C_3.0_N-Se_0.03_ in comparison with other materials.

### Mechanism investigation on the performance of C_3.0_N-Se_0.03_

XPS spectra were analyzed deeply to identify the mechanism on MA selectivity over the prepared C_*x*_N-Se_*y*_ materials. It has been well accepted that the graphitic N species play significant role on oxidations when using N-doped carbon materials as the catalysts.^20^ Based on the high-resolution XPS spectra of N 1s (Fig. S11†), the content of the graphitic N species in the C_*x*_N-Se_*y*_ materials (Table S3†) decreased with the increase of Se content. However, although the content of graphitic N in C_3.0_N-Se_0.03_ was lower than those in C_0.7_N-Se_0.002_ and C_0.9_N-Se_0.003_, the MA selectivity over C_3.0_N-Se_0.03_ was higher than those over C_0.7_N-Se_0.002_ and C_0.9_N-Se_0.003_. This is because that more active sites could not only promote the oxidation of furfural to generate MA but also enhance the excessive oxidation of furfural. Meanwhile, C_4.2_N-Se_0.05_ had the lowest content of active graphitic N, which may result in some intermediates being unoxidized over C_4.2_N-Se_0.05_, and thus, the selectivity of MA over C_4.2_N-Se_0.05_ was lower than that over C_3.0_N-Se_0.03_. On the basis of the above discussion, C_3.0_N-Se_0.03_ with suitable content of graphitic N provided the highest MA selectivity. In another aspect, the binding energy of graphitic N in the C_3.0_N-Se_0.03_ was higher than those in other four C_*x*_N-Se_*y*_ materials, indicating that the graphitic N in the C_3.0_N-Se_0.03_ was more positively charged. The more positively charged graphitic N would result in the *ortho*-carbon of the graphitic N being more positive, which was helpful for the formation of active oxygen species on this carbon.^21^ This result further explained the higher catalytic activity of C_3.0_N-Se_0.03_ among the synthesized five C_*x*_N-Se_*y*_.

Besides, all the C_*x*_N-Se_*y*_ materials showed better catalytic performance than g-C_3_N_4_ because the different chemical structures of C_*x*_N-Se_*y*_ and g-C_3_N_4_ (owing to the doping of Se) resulted in the different mechanism. Generally, g-C_3_N_4_ acted as the electron donor to activate O_2_ to form 
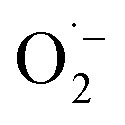
.^22^ However, the rate of this process was slow, and the generated 
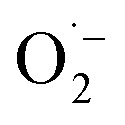
 was not a selective species. In contrast, over C_*x*_N-Se_*y*_, the oxygen radical was directly formed on the materials, which was much faster than that of g-C_3_N_4_, and the obtained oxygen radical was more selective due to the satirical effect of the solid materials. Thus, both the activity and selectivity over g-C_3_N_4_ were higher than those over g-C_3_N_4_.

Additionally, Se doping was indispensable for the excellent performance of C_3.0_N-Se_0.03_ nanotubes. Although Se was not the direct catalytic site, the Se doping played the role of adjusting the amount and charge delocalization of the active site (graphitic N), which could contribute to the activity and selectivity for the electro-oxidation of furfural into MA. Thereby, all C_*x*_N-Se_*y*_ materials showed better performance than g-C_3_N_4_, and C_3.0_N-Se_0.03_ with suitable amount and higher charge delocalization was the best among all the examined materials.

### Scope of the substrates

Inspired by the excellent performance of C_3.0_N-Se_0.03_ with furfural as the substrate, we explored the possibility of MA synthesis from electrochemical oxidation of other biomass-derived furan compounds over C_3.0_N-Se_0.03_ (Table 1). It was found that C_3.0_N-Se_0.03_ could catalyze the electro-oxidation of various furan compounds to MA. More importantly, the substituent groups significantly affected the yield of MA (Table 1, entries 1–3), which increased following the order: furfuryl alcohol (21.1%) < furfuryl (84.2) < furoic acid (95.2%). This might be resulted from that furfuryl alcohol needed most steps to obtain MA, which increased the possibility of side-reactions. A similar tendency was observed when using 5-hydroxymethylfurfural and its derivatives as the substrates (Table 1, entries 4–8). The yields of MA could reach 95.2% and 90.7% when furoic acid and 2,5-furandicarboxylic acid were employed as the reactants (Table 1, entries 3 and 5), respectively. These results indicated the general applicability of the C_3.0_N-Se_0.03_ on the synthesis of MA from the electrocatalytic oxidation of biomass-derived furan compounds. From the view point of practical application, more works should be conducted, such as the influence of impurity on the reaction.

**Table 1:** Electrochemical oxidation of other reactants over the C_3.0_N-Se_0.03_^a^

Entry	Reactant	C (%)	Y (%)	S (%)
1	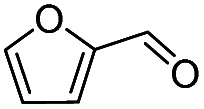	99.5	84.2	84.0
2	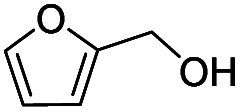	93.6	21.2	22.6
3	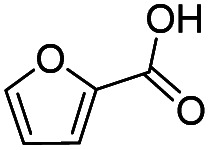	100	95.2	95.2
4	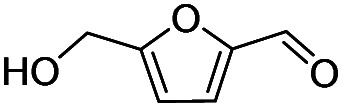	92.8	49.3	53.1
5	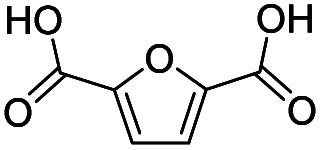	96.3	90.7	94.2
6	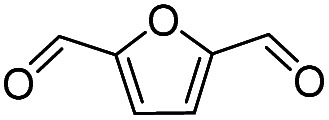	100	42.1	42.1
7	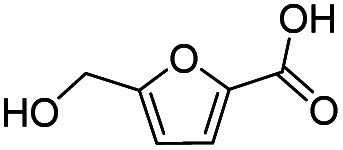	99.7	67.8	68.0
8	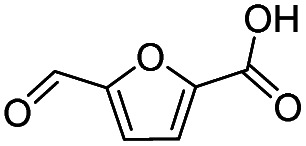	99.3	56.9	57.3

a Reaction conditions: applied potential, 1.7 V *vs.* Ag/AgCl; reactant concentration, 10 mM; 0.5 M aqueous KHCO_3_ electrolyte, 15 mL; reaction time, 6 h.

## Conclusions

In conclusion, metal-free graphitic carbon nitride nanotubes, C_3.0_N-Se_0.03_, have been fabricated by pyrolysis of urea, NH_4_Cl, and SeO_2_. As the metal-free catalyst, C_3.0_N-Se_0.03_ showed excellent performance for the electrocatalytic oxidation of various biomass-derived furan compounds into MA. The yield of MA could reach 84.2% from furfural oxidation at an applied potential of 1.7 V *vs.* Ag/AgCl. Moreover, it was observed that the substituent groups on the furan ring significantly affected the reaction selectivity. The substrates with COOH group generally provided higher yields of MA, and the yield of MA from FA and 2,5-furandicarboxylic acid could reach 95.2% and 90.7%, respectively. More importantly, the performance of C_3.0_N-Se_0.03_ was much better than that of g-C_3_N_4_ and other C_*x*_N-Se_*y*_ materials, which was caused by the higher electrochemical surface area and lower charge transfer resistance. This work provides an efficient and selective route to synthesize MA from biomass-derived furan compounds using metal-free electrocatalyst.

## Experimental

### Materials

Furfural (99%), furan (98%) and 5-formyl-2-furancarboxylic acid (FFCA, >98%) were purchased from Aladdin. 5-Hydroxymethylfurfural (HMF, 98%) was purchased from Sigma Aldrich Co. Ltd. 2,5-Furandicarboxylic acid (FDCA, 97%) and furfuryl alcohol (98%) were purchased from Alfa Aesar China Co., Ltd. 2,5-Diformylfuran (DFF, 98%) and 5-hydroxymethyl-2-furancarboxylic acid (HMFCA, 98%) were purchased from J&K Scientific Ltd. SeO_2_ (99%), urea (99%), 2-furoic acid (98%) and 2(5*H*)-furanone (98%) were purchased from Innochem Scientific Ltd. NH_4_Cl (≥99.5%) was purchased from Beijing Chemical Works. Toray carbon paper (CP, TGP-H-60, 19 × 19 cm), Nafion D-521 dispersion (5% w/w in water and 1-propanol, ≥ 0.92 meg g^−1^ exchange capacity) and Nafion N-117 membrane (0.180 mm thick, ≥0.90 meg g^−1^ exchange capacity) were purchased from Alfa Aesar China Co., Ltd. All reagents purchased from commercial sources were used as obtained without further purification.

### Preparation of C_*x*_N-Se_*y*_

The route to synthesize different C_*x*_N-Se_*y*_ was very similar except for the different usage of SeO_2_. Herein, we described the route for the preparation of C_3.0_N-Se_0.03_ as an example. Typically, NH_4_Cl, SeO_2_ and urea with a mass ratio of 10 : 1 : 1 was added into a polytetrafluoroethylene milling jar along with agate balls. Afterwards, the jar was placed in a vibrating ball miller (300 r min^−1^) and the mixture was ball-milled for 6 h. The obtained white product was dried and then carbonized in a tube furnace at 350 °C for 2 h and 550 °C for 2 h under Ar atmosphere (at a heating rate of 5 °C min^−1^). The obtained black powder was collected after the temperature was cooled down to room temperature. The C/N atomic ratio was detected to be 3.0 by element analysis, and the Se content was 2.39 wt% determined by ICP-AES.

Other C_*x*_N-Se_*y*_ materials with different mass ratios of SeO_2_ and urea (0.1, 0.25, 0.5 and 1.5) were synthesized, and the corresponding C/N atomic ratio was detected to be 0.7, 0.9, 2.1, and 4.2 by element analysis, respectively. Meanwhile, determined by ICP-AES, the Se contents in these C_*x*_N-Se_*y*_ materials were 0.68 wt%, 0.82 wt%, 1.26 wt% and 3.46 wt%, respectively. Thus, the obtained materials were named as C_0.7_N-Se_0.002_, C_0.9_N-Se_0.003_, C_2.1_N-Se_0.01_, and C_4.2_N-Se_0.05_, according to the C/N and Se/N atomic ratios of the final products.

### Preparation of g-C_3_N_4_-AC

The route for the synthesis of g*-*C_3_N_4_-AC was the same as that for the preparation of C_*x*_N-Se_*y*_ except for the absence of SeO_2_.

### Preparation of bulk g-C_3_N_4_

The route for the synthesis of bulk g*-*C_3_N_4_ was the same as that for the preparation of g*-*C_3_N_4_-AC except that only urea was used.

### Characterization

Powder X-ray diffraction (XRD) patterns were collected on the X-ray diffractometer (Model D/MAX2500, Rigaku) with Cu-Kα radiation. X-ray photoelectron spectroscopy (XPS) analysis was performed on the Thermo Scientific ESCA Lab 250Xi using 200 W monochromatic Al Kα radiation, and the 500 μm X-ray spot was used. The base pressure in the analysis chamber was about 3 × 10^−10^ mbar. Typically, the hydrocarbon C 1s line at 284.8 eV from adventitious carbon was used for energy referencing. The morphologies of materials were characterized by a HITACHI S-4800 scanning electron microscope (SEM) and a JEOL JEM-2100F high-resolution transmission electron microscopy (HR-TEM). The samples were dispersed in ethanol using an ultrasonic bath, and the final suspensions were transferred to TEM grids and dried in ambient air before electron microscopy analysis. N_2_ adsorption/desorption isotherms of the materials were measured on a Quadrasorb SI-MP system at 77 K. Before analysis, samples were allowed to outgas at 180 °C under turbomolecular vacuum pumping for a minimum of 5 h. Raman spectroscopy was carried out using LabRAM HR Evolution. Fourier transform infrared (FT-IR) spectra were obtained using a Bruker Tensor 27 spectrometer, and the sample was prepared by KBr pellet method. X-ray absorption fine structure (XAFS) measurement for Se K-edge was carried out at room temperature at the 1W1B beamline of the Beijing Synchrotron Radiation Facility (BSRF). For XAFS analysis, the extraction of the *χ*(*k*) function was performed using the Athena program. Prior to merging, the spectrum was aligned to the first and largest peak in the smoothed first derivative of the absorption spectrum, background removed, and normalized. The averaged *k*^3^-weight *χ*(*k*) function was Fourier transformed with a Rbkg value of 1.0. Normalized *μ*(*E*) data were obtained directly from the Athena program of the IFEFFIT package.

### Preparation of electrode

To prepare the electrode, 2 mg of the as-prepared catalysts and 10 μL of Nafion D-521 dispersion were dispersed in 1 mL acetone and ultrasonicated to form uniform suspension, and suitable amount of the suspension was loaded on the 1 cm × 1 cm CP. After being dried in N_2_ atmosphere, the loading of catalyst could be calculated as 2 mg cm^−2^ on the basis of the CP's weight change.

### Linear sweep voltammetry (LSV) measurement

An electrochemical workstation (CHI 660E, Shanghai CH Instruments Co., China) was employed. Linear sweep voltammetry (LSV) measurements were conducted in a single compartment cell with three electrodes, including a working electrode, a platinum gauze auxiliary electrode, and an Ag/AgCl reference electrode. The LSV measurements in electrolyte were carried out in a certain potential range at a sweep rate of 50 mV s^−1^ under slight magnetic stirring. In addition, aqueous KHCO_3_, H_2_SO_4_, KOH, (NH_4_)_2_SO_4_ solutions and phosphate buffer solution (PBS, pH = 7) were chose as the electrolyte (both anolyte and catholyte) to study the optimal reaction conditions for electrochemical oxidation of furfural to maleic acid.

### Electrochemical oxidation of furfural

The electrochemical oxidation of furfural was carried out at room temperature using an electrochemical workstation (CHI 660E, Shanghai CH Instruments Co., China) with a typical H-type cell, which was separated by a Nafion membrane. The electrochemical cell is configured with a three-electrode system: a working electrode as anode, a platinum gauze auxiliary electrode as the counter electrode (cathode), and an Ag/AgCl reference electrode. The best electrochemical oxidation experiment was conducted in 15 mL of a 0.5 M KHCO_3_ solution in the presence or absence of 10 mM furfural.

### HPLC analysis of oxidation products

The conversion of furfural and the yield of the corresponding products were characterized by HPLC technique. Specifically, 100 μL samples were collected from the electrolyte after the addition of furfural and at different reaction time and then diluted with 900 μL water. After filtrated by a 0.22 nm filter, the samples were used for the HPLC analysis. The HPLC analysis was conducted on a Shimadzu Prominence Liquid Chromatograph (LC-20TA) equipped with a C18 column (Ascentis Express, 15 cm × 2.1 mm, 2.7 μm) and an ultraviolet detector. Mobile phase A is 0.1% formic acid aqueous solution adjusted to pH = ∼3 with ammonium formate, and mobile phase B was acetonitrile. The flow rate is 0.3 mL min^−1^ (gradient program: 100% A for 2 min, to 20% A over 32 min, and held for 18 min). The quantification of furfural and its oxidation products were calculated based on the calibration curves of those standard compounds purchased from commercial vendors.

## Author contributions

Xin Huang, Jinliang Song and Buxing Han proposed the project, designed and conducted the experiments and wrote the manuscript. Other authors performed some experiments and discussed the work.

## Conflicts of interest

There are no conflicts to declare.

## Supplementary Material

SC-012-D1SC01231B-s001
