# Supplementary material for: Robust selenium-doped carbon nitride nanotubes for selective electrocatalytic oxidation of furan compounds to maleic acid
Source: Chem Sci. 2021 Apr 1;12(18):6342–9. doi: 10.1039/d1sc01231b (PMC8115246; doi:10.1039/d1sc01231b)
Supplement: SC-012-D1SC01231B-s001 [file SC-012-D1SC01231B-s001.pdf]

Electronic Supplementary Material (ESI) for Chemical Science.
This journal is © The Royal Society of Chemistry 2021

Supporting information for:

Robust selenium-doped carbon nitride nanotubes for selective electrocatalytic oxidation of furan compounds to maleic acid

Xin Huang,[a,b] Jinliang Song,[a,c] Manli Hua,[a,b] Bingfeng Chen,[a] Zhenbing Xie,[a,b] Huizhen Liu,[a,b,c] and Zhanrong Zhang,[a] Qinglei Meng,[a,c] and Buxing Han[a,b,c*]*

[a]Beijing National Laboratory for Molecular Sciences, CAS Key Laboratory of Colloid, Interface and Chemical Thermodynamics, CAS Research/Education Center for Excellence in Molecular Sciences, Institute of Chemistry, Chinese Academy of Sciences, Beijing 100190, China.
[b]School of Chemistry and Chemical Engineering, University of Chinese Academy of Sciences, Beijing 100049, China.
[c]Physical Science Laboratory, Huairou National Comprehensive Science Center, Beijing 101400, China.
E-mails: songjl@iccas.ac.cn, hanbx@iccas.ac.cn

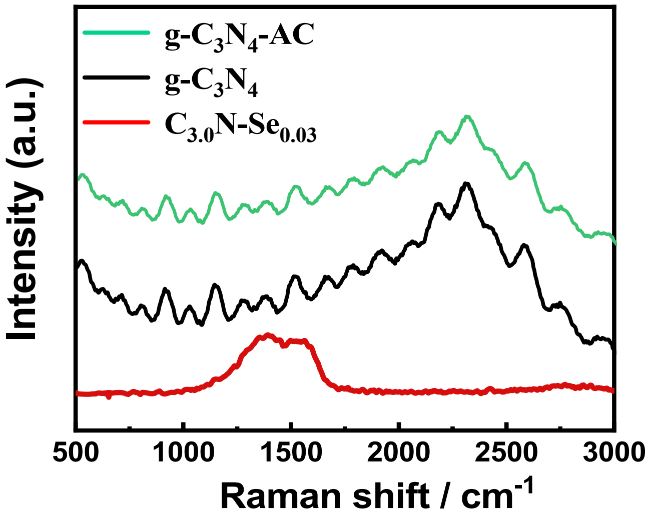

Fig. S1. Raman spectra of the prepared g-C_3N_4, g-C_3N_4-AC and $C_{3.0}N$-$Se_{0.03}$. The g-C_3N_4 and g-C_3N_4-AC only showed some characteristic peaks in the region of 500-3000 cm^{-1}, which corresponded to the stretching vibrations of heptazine heterocyclic ring units in their 2D conjugated framework.

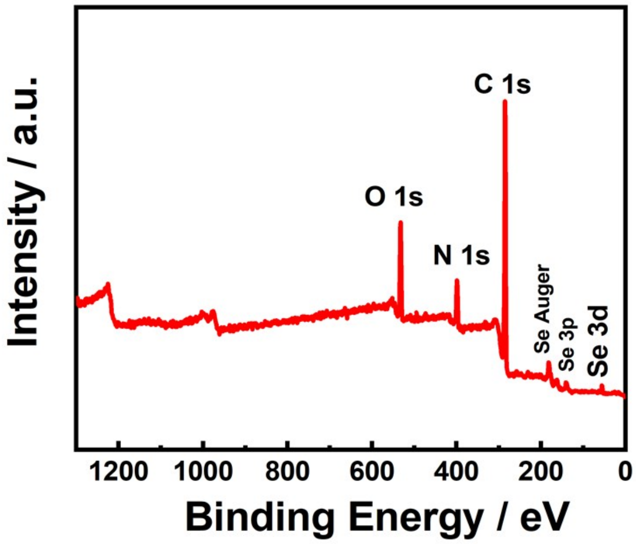

Fig. S2. XPS survey spectra of $C_{3.0}N$-$Se_{0.03}$.

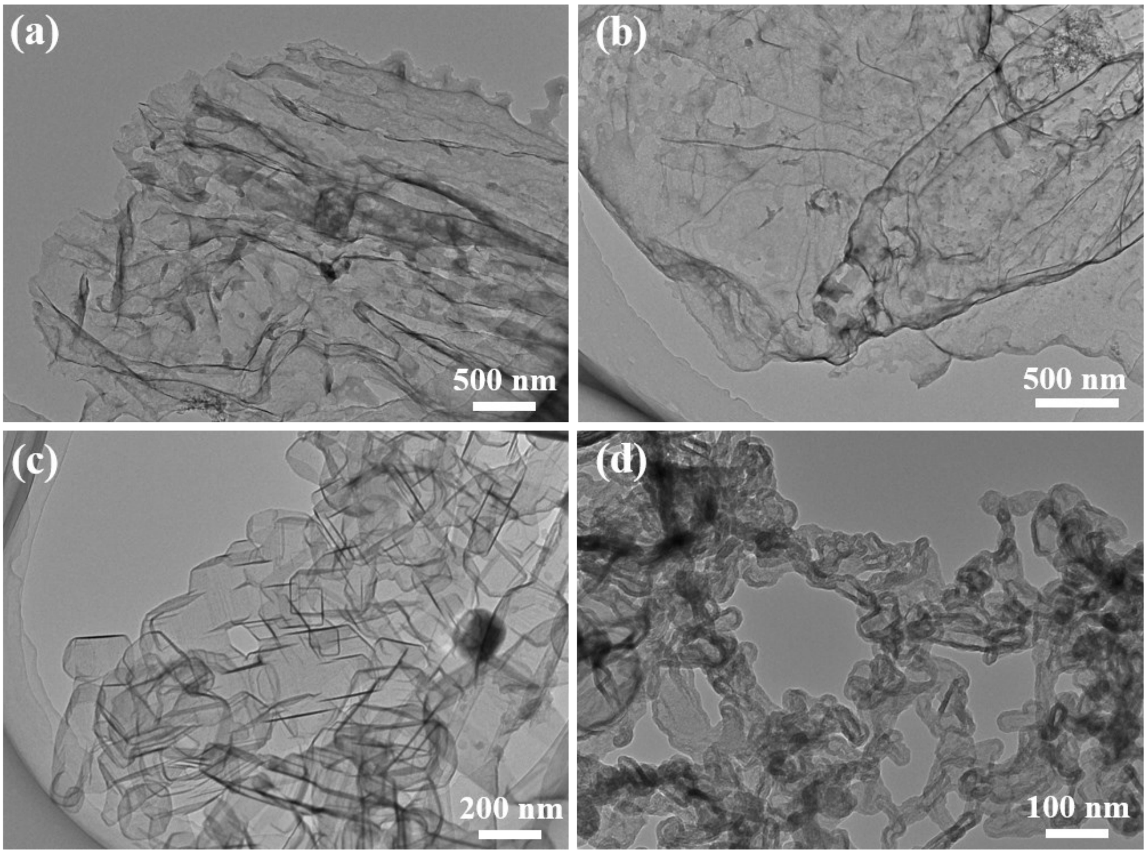

Fig. S3. TEM images of the prepared other C_xN-Se_y materials. (a) $C_{0.7}N$-$Se_{0.002}$, (b) $C_{0.9}N$-$Se_{0.003}$, (c) $C_{2.1}N$-$Se_{0.01}$, and (d) $C_{4.2}N$-$Se_{0.05}$. The morphology of the C_xN-Se_y materials with different C/N ratios was significantly different. $C_{0.7}N$-$Se_{0.002}$ and $C_{0.9}N$-$Se_{0.003}$ had a thin nanosheet structure (Fig. S3a and b). Some portions of $C_{2.1}N$-$Se_{0.01}$ material possessed the hollow column structure (Fig. S3c), while $C_{4.2}N$-$Se_{0.05}$ showed curved tubular structure with width in the range of about 20 to 50 nm (Fig. S3d). In comparison with the $C_{3.0}N$-$Se_{0.03}$, the hollow cavity width of $C_{4.2}N$-$Se_{0.05}$ was smaller and the curvature and agglomeration of tubes were more serious.

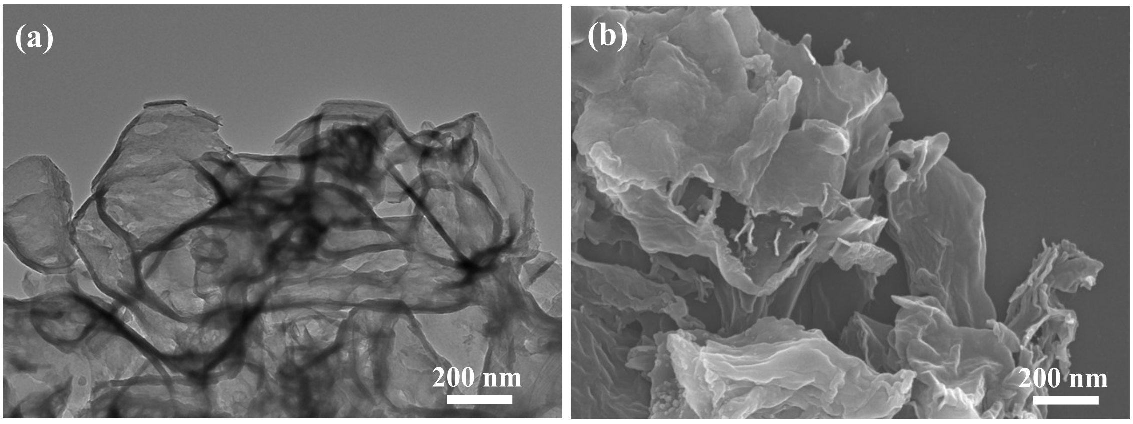

Fig. S4. (a) TEM image and (b) SEM image of the prepared *g*-C_3N_4

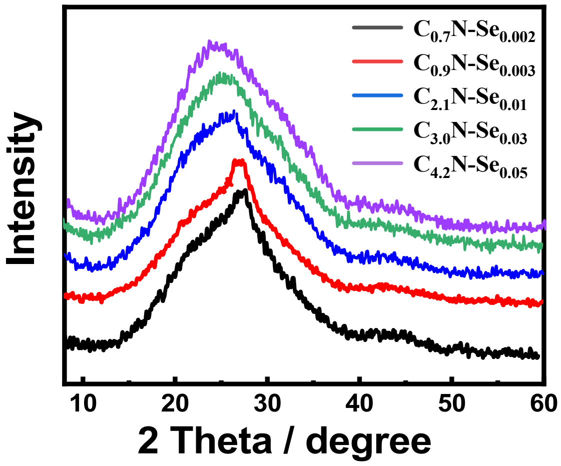

Fig. S5. XRD patterns of $C_{0.7}N\text{-}Se_{0.002}$, $C_{0.9}N\text{-}Se_{0.003}$, $C_{2.1}N\text{-}Se_{0.01}$, $C_{3.0}N\text{-}Se_{0.03}$, and $C_{4.2}N\text{-}Se_{0.05}$. $C_{0.7}N\text{-}Se_{0.002}$ and $C_{0.9}N\text{-}Se_{0.003}$ showed signals of both conjugated tri-s-triazine and graphene-like turbostratic forms, while the typical ordering turbostratic form was observed for the $C_{2.1}N\text{-}Se_{0.01}$, $C_{3.0}N\text{-}Se_{0.03}$ and $C_{4.2}N\text{-}Se_{0.05}$. Moreover, a gradual decrease of interlayer distance could be found when more Se was incorprated in the framework.

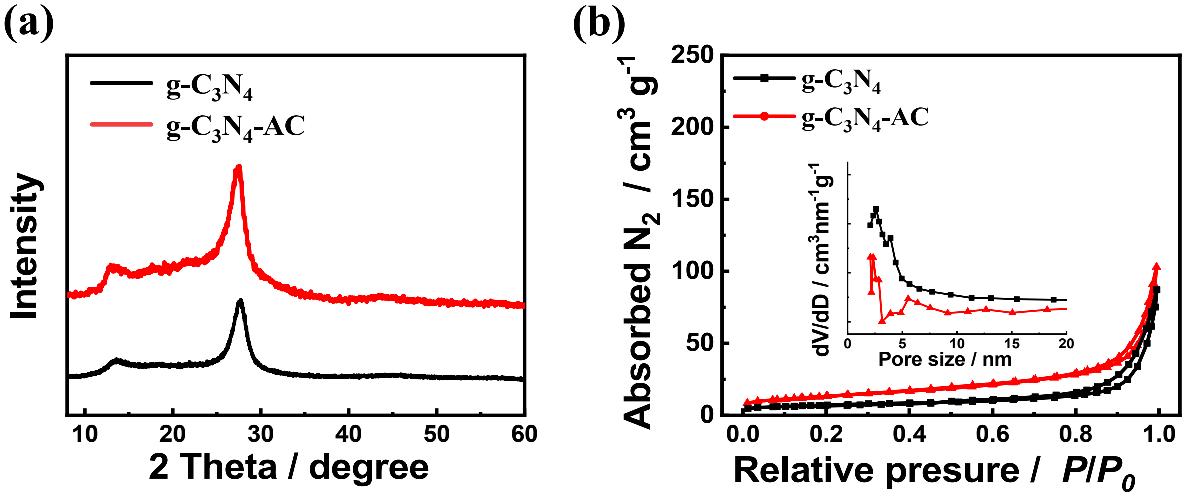

Fig. S6. (a) XRD patterns, and (b) N_2 adsorption-desorption isotherms of *g*-C_3N_4 (synthesized from urea) and *g*-C_3N_4-AC (synthesized from urea and NH_4Cl). A slight shift of single broad diffraction peak at 24.8° the $C_{3.0}N\text{-}Se_{0.03}$ (1e) compared with the typical peak (27.8°) in *g*-C_3N_4 (synthesized from urea) and *g*-C_3N_4-AC (synthesized from urea and NH_4Cl) can be found, implying the decrease of interlayer distance in $C_{3.0}N\text{-}Se_{0.03}$ caused by Se doping. Meanwhile, the peak centered at 13.0° in *g*-C_3N_4 and *g*-C_3N_4-AC, which was the characteristic peak for the tri-striazine structure, was not obsereved in $C_{3.0}N\text{-}Se_{0.03}$, indicating the change of the tri-s-triazine structure owing to the Se doping and the different C/N atomic ratio.

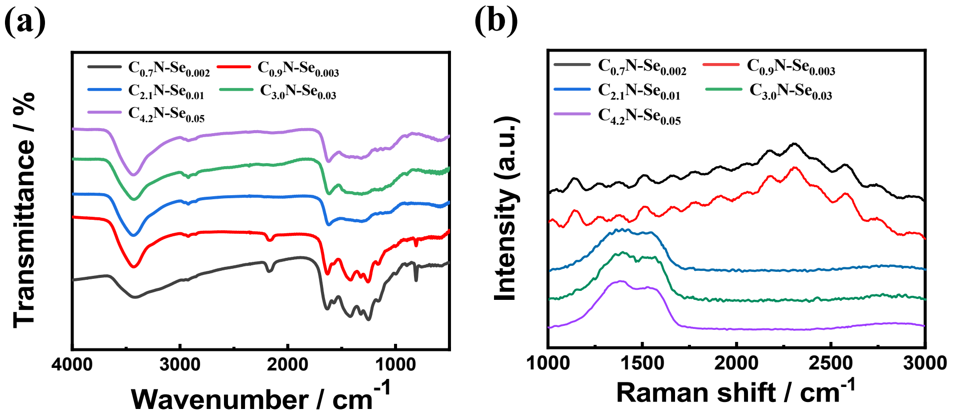

Fig. S7. FT-IR spectra (a) and Raman spectra (b) of $C_{0.7}N$-$Se_{0.002}$, $C_{0.9}N$-$Se_{0.003}$, $C_{2.1}N$-$Se_{0.01}$, $C_{3.0}N$-$Se_{0.03}$, and $C_{4.2}N$-$Se_{0.05}$. With increasing C/N atomic ratio, the band at 810 cm^{-1} (ascribed to tritazine rings) in FT-IR spectra became weaker (Fig. S7a), and the graphene-like structure of C_xN-Se_y materials became stronger (Fig. S7b). $C_{2.1}N$-$Se_{0.01}$, $C_{3.0}N$-$Se_{0.03}$ and $C_{4.2}N$-$Se_{0.05}$ had similar FT-IR and Raman spectra, implying the very close structure of these three materials.

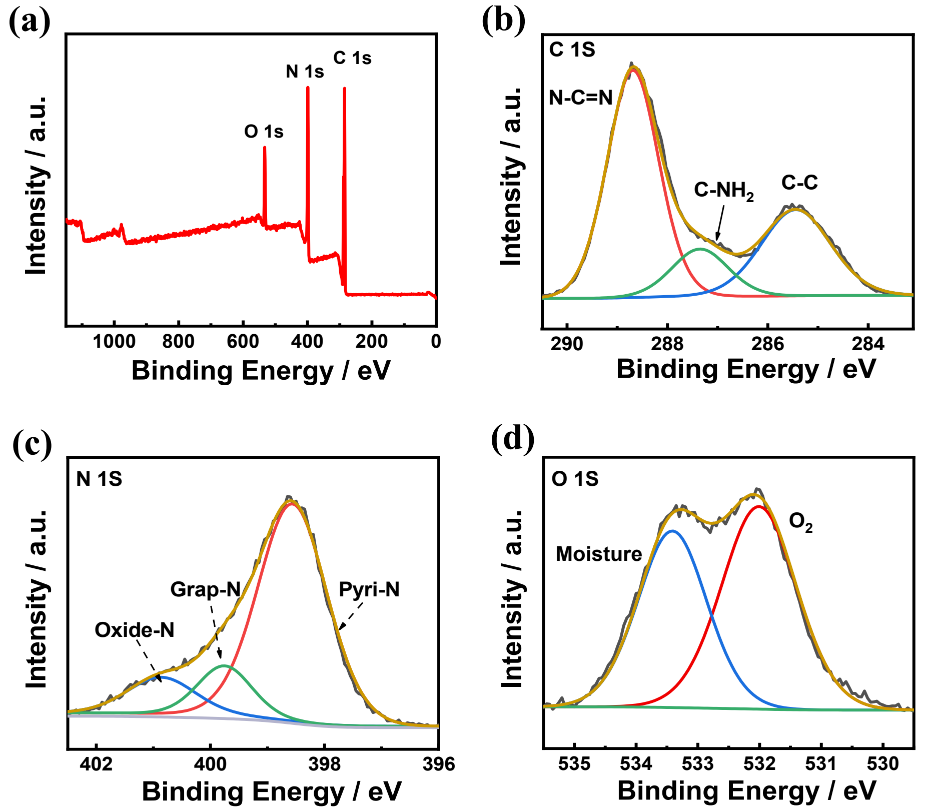

Fig. S8. XPS spectra of the prepared g-C_3N_4. (a) XPS survey spectra, (b) high-resolution XPS spectra of C 1s, (c) high-resolution XPS spectra of N 1s, and (d) high-resolution XPS spectra of O 1s. The strength of C-C and N-$(C)_3$ bonds in $C_{3.0}N$-$Se_{0.03}$ was significantly enhanced compared with those in g-C_3N_4, which may be caused by the high content of C atoms in $C_{3.0}N$-$Se_{0.03}$.

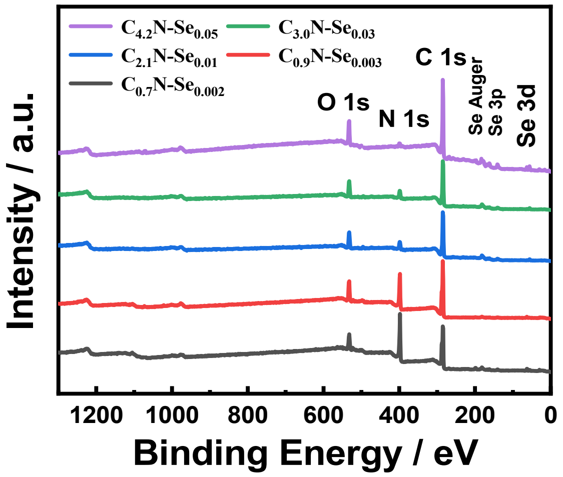

Fig. S9. XPS survey spectra of $C_{0.7}N\text{-}Se_{0.002}$, $C_{0.9}N\text{-}Se_{0.003}$, $C_{2.1}N\text{-}Se_{0.01}$, $C_{3.0}N\text{-}Se_{0.03}$, and $C_{4.2}N\text{-}Se_{0.05}$. The signals for Se and C became stronger with the increase of the used SeO_2 amount, while the N signals became weaker.

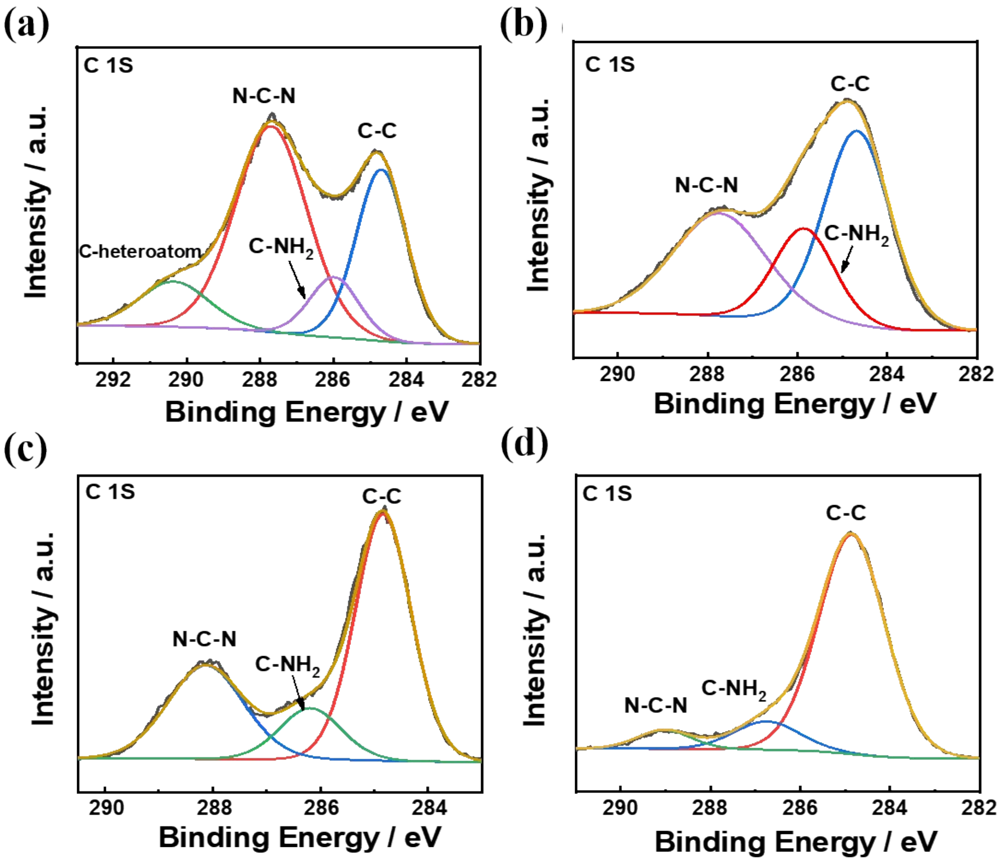

Fig. S10. High-resolution XPS spectra of C 1s. (a) $C_{0.7}N\text{-}Se_{0.002}$, (b) $C_{0.9}N\text{-}Se_{0.003}$, (c) $C_{2.1}N\text{-}Se_{0.01}$, and (d) $C_{4.2}N\text{-}Se_{0.05}$. The content of sp^2 C-C bond was obviously increased with the increasing amount of the used SeO_2, while the content of sp^2 C-N-C decreased.

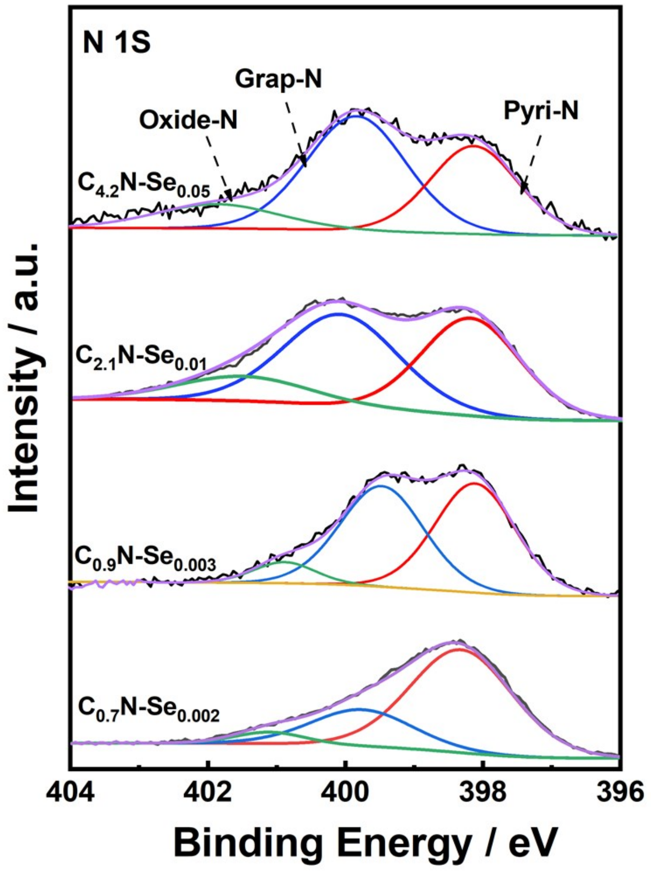

Fig. S11. High-resolution XPS spectra of N 1s for $C_{0.7}N\text{-}Se_{0.002}$, $C_{0.9}N\text{-}Se_{0.003}$, $C_{2.1}N\text{-}Se_{0.01}$, and $C_{4.2}N\text{-}Se_{0.05}$. XPS spectra of N 1s showed that with the increase of Se content, the ratio of graphitic N obviously increased while an opposite tendency was found for pyridinic N.

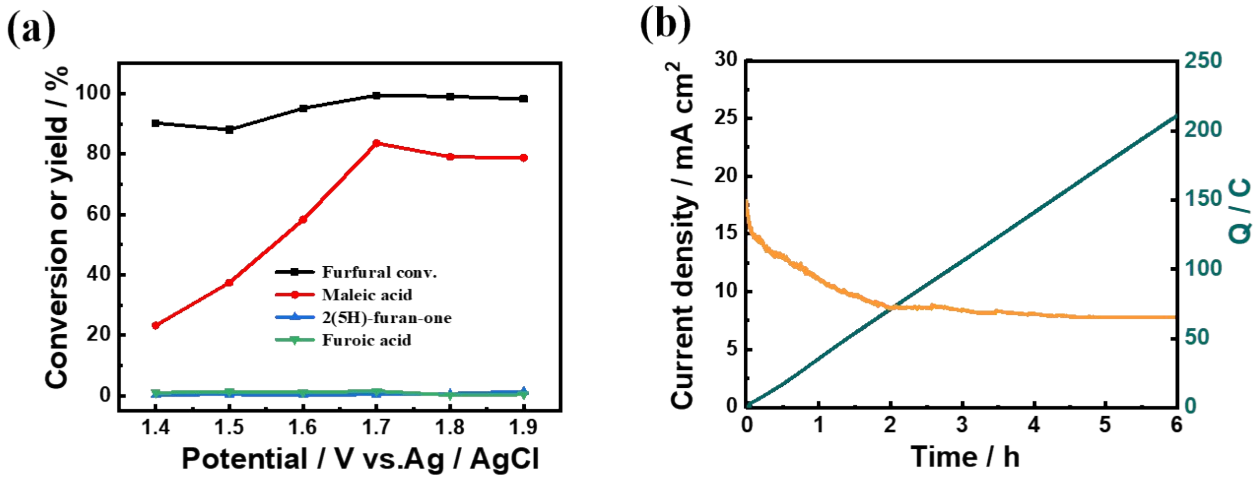

Fig. S12. (a) Conversion of furfural and yields of various products at different applied potentials over $C_{3.0}N\text{-}Se_{0.03}$ in 0.5 M $KHCO_3$ solution, and (b) Current density and total charge at different electrolysis times at 1.7 V *vs.* Ag/AgCl.

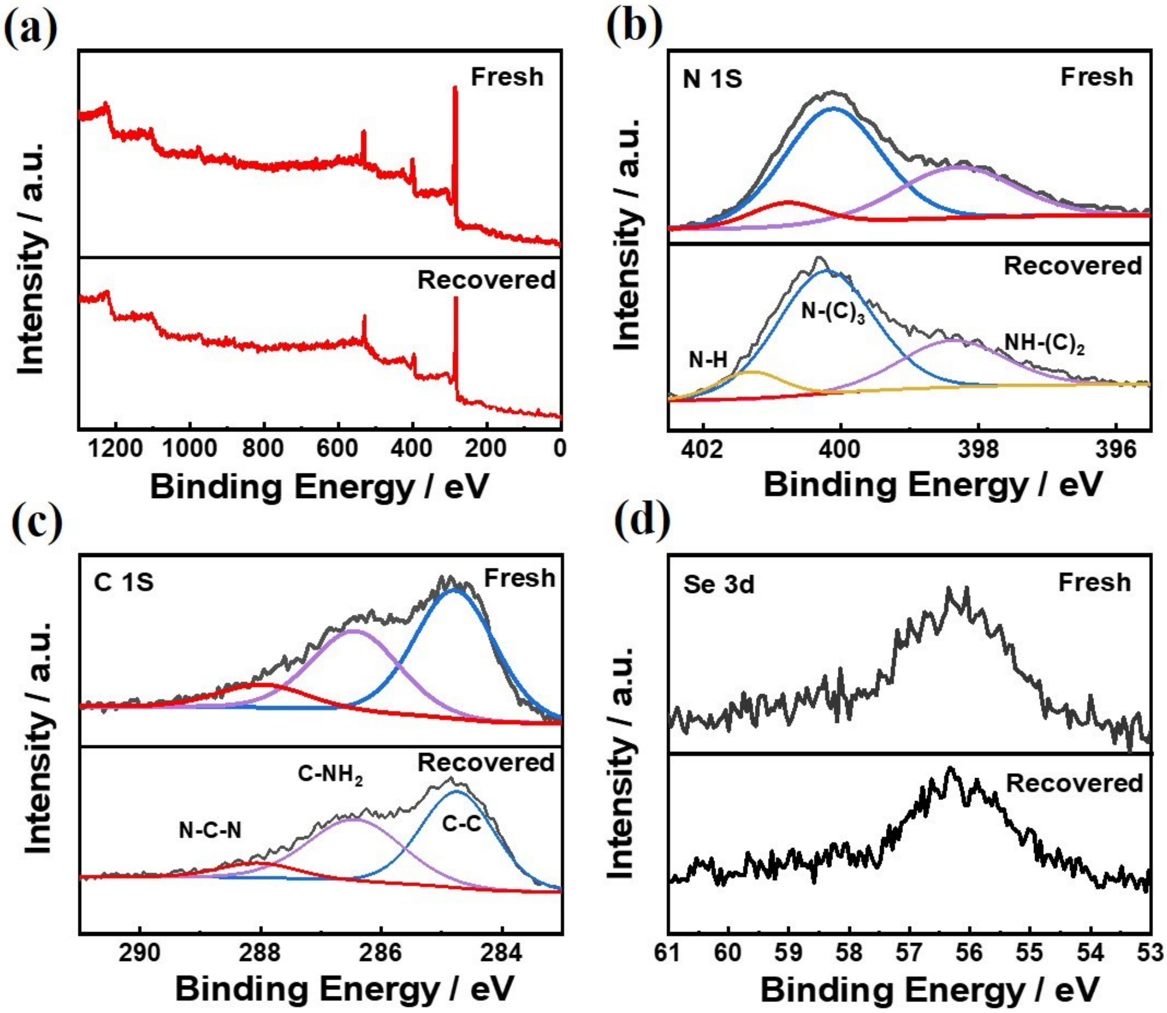

Fig. S13. XPS spectra of fresh and recovered $C_{3.0}N\text{-}Se_{0.03}$.

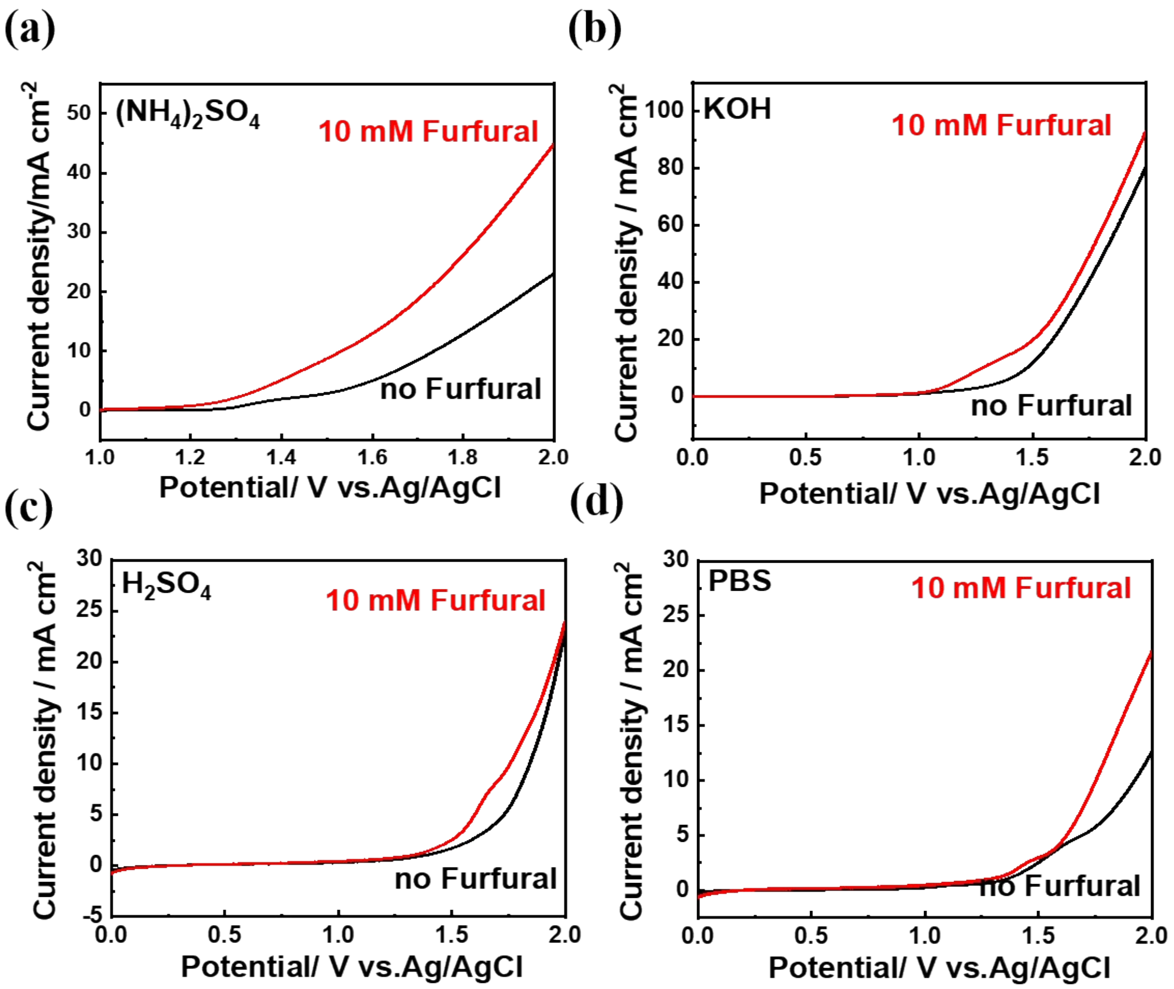

Fig. S14. LSV curves at a scan rate of 50 mV/s with and without 10 mM Furfural in (a) 0.5 M $(NH_4)_2SO_4$, (b) 1 M of KOH, (c) 1M H_2SO_4, and (D) pH=7 phosphate buffered solution (PBS).

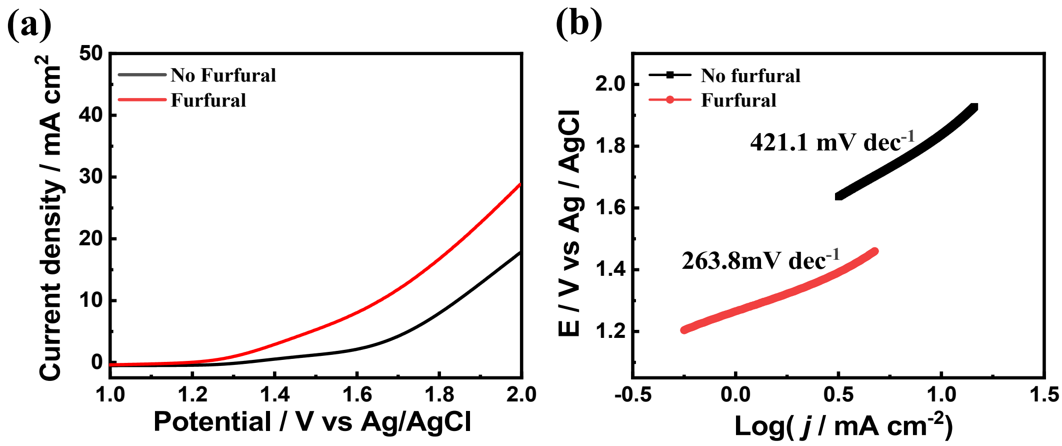

Fig. S15. (a) LSV curves of *g*-C_3N_4 at a scan rate of 50 mV/s, and (b) Tafel plots of *g*-C_3N_4 with and without furfural in 0.5 M aqueous $KHCO_3$ solution.

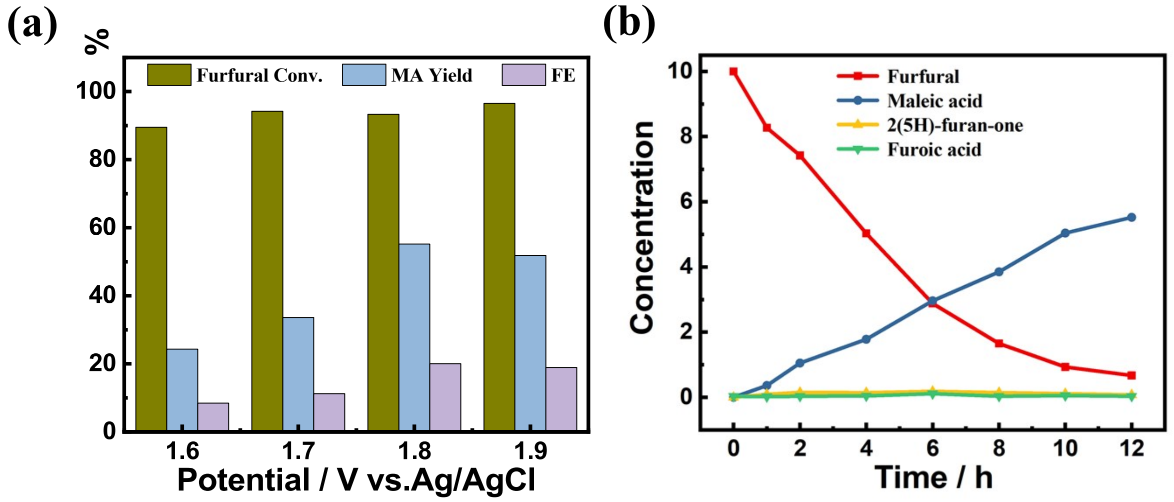

Fig. S16. (a) The furfural conversion, MA yield and FE at different applied potentials, and (b) Concentrations of furfural and the oxidation products at various electrolysis times over pure *g*-C_3N_4 electrode at 1.8 V *vs.* Ag/AgCl in 0.5 M aqueous $KHCO_3$ electrolyte.

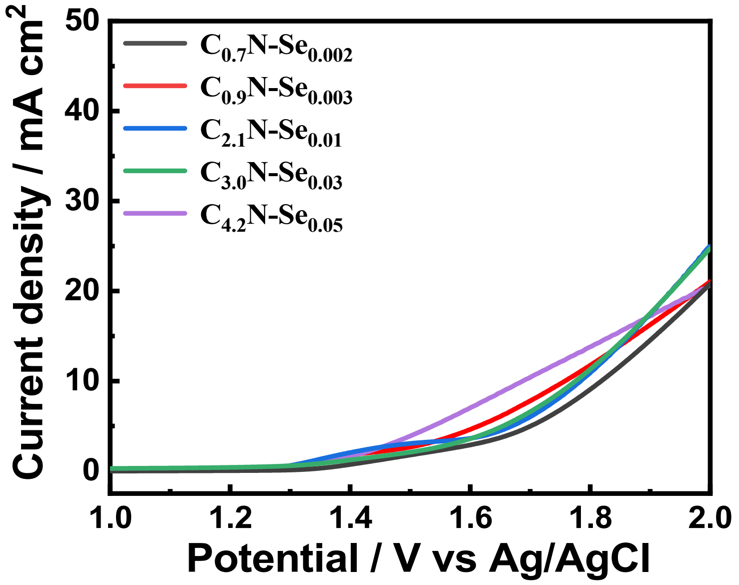

Fig. S17. LSV curves of a serious of C_xN-Se_y materials at a scan rate of 50 mV/s without furfural in 0.5 M $KHCO_3$ solution.

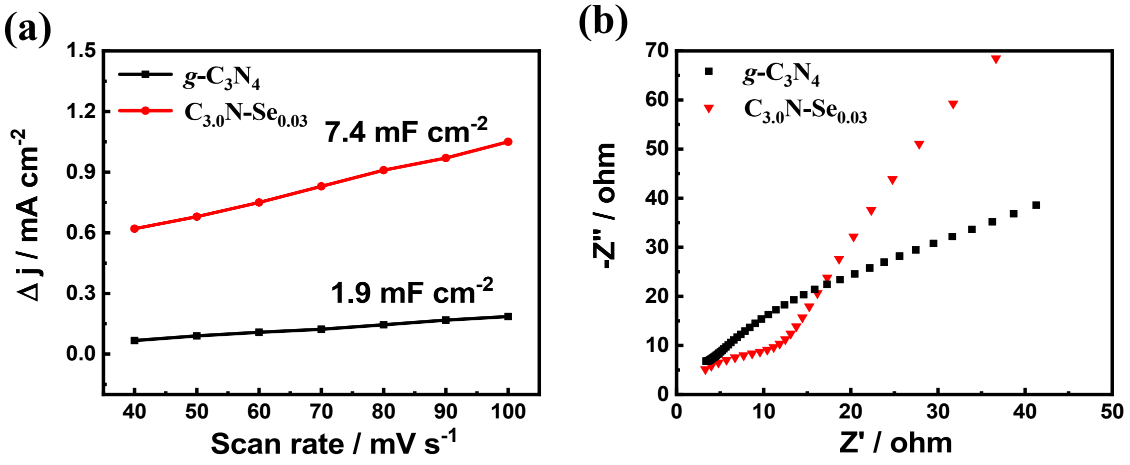

Fig. S18. (a) Charging current density differences plotted against scan rates, and (b) Nyquist plots of *g*-C_3N_4 and $C_{3.0}N$-$Se_{0.03}$ in 0.5 M $KHCO_3$ solution at an open circuit potential.

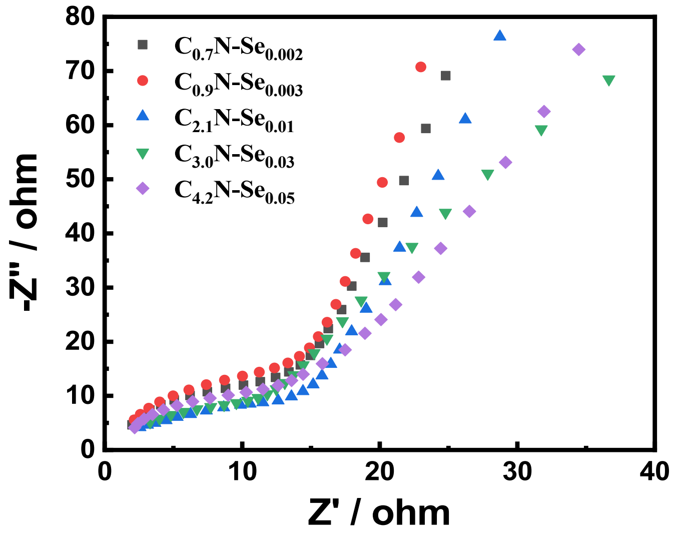

Fig. S19. Nyquist plots of various C_xN-Se_y electrodes in 0.5 M $KHCO_3$ solution at an open circuit potential.

Table S1. Furfural oxidation using various heterogeneous catalysts.

Catalysts	Furfural conv. (%)	MA yield (%)	MA selec. (%)	References
$C_{3.0}N$-$Se_{0.03}$	99.5	84.2	84.0	This work
Amberlyst-15	＞99	11	11	1
Nb_2O_5	＞99	5	5	1
ZrO_2	＞99	5	5	1
ZSM-5	＞99	2.4	2.4	2
$H_5PV_2Mo_{10}O_{40}\cdot xH_2O$	98.7	54	55	3
$H_5PV_2Mo_{10}O_{40}\cdot xH_2O$	100	41.8	41.8	4
TS-1	100	62	62	5
V_2O_5/SnO_2	74.4	38.9	52.3	6
FeT(*p*-Br)PPCl/SBA-15	53.7	38	70.8	7

Table S2. Electrochemical oxidation of furfural and distribution of products over $C_{3.0}N\text{-}Se_{0.03}$ in various electrolytes with different acidity and alkalinity.

Eletrolytes	pH	Potential (V)	Furfural conversion (%)	MA yield (%)	Furanone yield (%)	FA yield (%)	Selectivity of MA (%)
0.5 M H_2SO_4	0.42	1.7	98.1	20.8	1.4	< 0.1	21.2
0.5 M $(NH_4)_2SO_4$	5.18	1.7	90.7	59.8	3.4	< 0.1	65.9
PBS（pH=7）	7.01	1.7	91.1	15.6	0.27	0.19	17.1
1 M KOH	13.67	1.6	100	37.8	1.1	2.65	37.8

Table S3. Concentration of surface nitrogen species of various electrodes.

Sample	Total N (atom%)	Pyridinic N (atom%)	Graphitic N (atom%)	Oxidized N (atom%)
g-C_3N_4	20.6	17.6	2.2	1.8
$C_{0.7}N\text{-}Se_{0.002}$	15.1	9.6	4.4	1.1
$C_{0.9}N\text{-}Se_{0.003}$	14.2	7.8	5.4	1.0
$C_{2.1}N\text{-}Se_{0.01}$	8.0	4.0	3.5	0.5
$C_{3.0}N\text{-}Se_{0.03}$	5.1	2.3	2.4	0.3
$C_{4.2}N\text{-}Se_{0.05}$	3.3	1.1	1.9	0.3

References:

1. H. Choudhary, S. Nishimura, K. Ebitani, *Appl. Catal. A: Gen.,* 2013, **458**, 55-62.
2. H. Choudhary, S. Nishimura, K. Ebitani, *Chem. Lett.,* 2012, **23**, 409-411.
3. J. Lan, Z. Chen, J. Lin, G. Yin, *Green Chem.,* 2014, **16**, 4351-4358.
4. J. Lan, J. Lin, Z. Chen, G. Yin, *ACS Catal.,* 2015, **5**, 2035-2041.
5. Y. Lou, S. Marinkovic, B. Estrine, W. Qiang, G. Enderlin, *ACS Omega,* 2020, **5**, 2561-2568.
6. P. M. Malibo, P. R. Makgwane, P. G. Baker, *ChemistrySelect,* 2020, **5**, 6255-6267.
7. Y. Xie, Y. Huang, C. Wu, W. Yuan, Y. Xia, X. Liu, H. Wang, *Mol. Catal.,* 2018, **452**, 20-27.
